# Supplementary material for: Identification of photoperiod-induced specific miRNAs in the adrenal glands of Sunite sheep (Ovis aries)
Source: Front Vet Sci. 2022 Jul 22;9:888207. doi: 10.3389/fvets.2022.888207 (PMC9354845; doi:10.3389/fvets.2022.888207)
Supplement: Supplementary file 1 [file Data_Sheet_1.ZIP › Supplementary file/miRDeep2 file.zip!/miRDeep2 file/Know_Structure/LP42A3/oar-mir-107.pdf]

miRBase precursor : oar-mir-107
Total read count : 5643
oar-miR-107 read count : 5637
remaining reads : 6

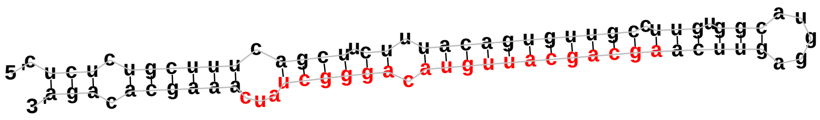

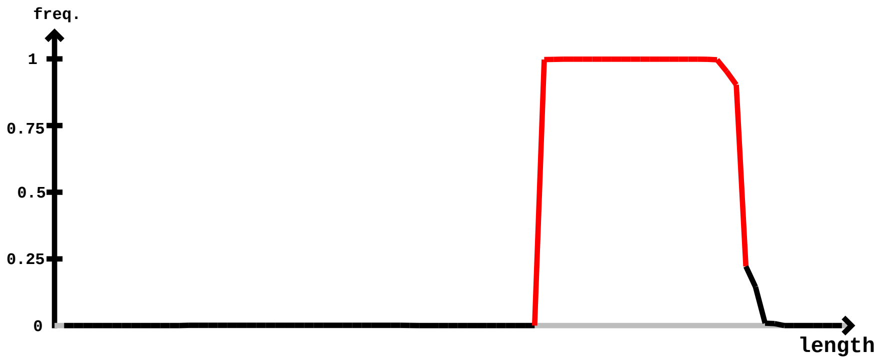


```
                                                 oar-miR-107
5'- cucucugcuuucagcuucuuuacaguguugccuuguggcauggaguucaagcagcauuguacagggcuaucaaagcacaga   -3'    exp
    .(((.((((((.((((.((.(((((((((((.(((.(((.....))))))))))))))))).))))))...)))))).)))    reads      mm         sample
    ............agcuucuuuacagugCugccuug..............................................    3          1          seq
    ............agcuucuuuacagugCugccuugu.............................................    3          1          seq
    .................................................agcagcauuguacaggg...............    3          0          seq
    .................................................agcagcauuguacagggc..............    10         0          seq
    .................................................agcagcauuguacagggcu.............    248        0          seq
    .................................................agcagcauuguacagggcua............    281        0          seq
    .................................................agcagcauuguacagggcuau...........    3838       0          seq
    .................................................agcagcauuguacagggcuauc..........    434        0          seq
    .................................................agcagcauuguacagggcuauca.........    768        0          seq
    .................................................agcagcauuguacagggcuaucaa........    9          0          seq
    .................................................agcagcauuguacagggcuauGaaa.......    36         1          seq
    .................................................agcagcauuguacagggcuaucaaa.......    2          0          seq
    ..................................................gcagcauuguacagggcuau...........    2          0          seq
    ..................................................gcagcauuguacagggcuauc..........    1          0          seq
    ...................................................cagcauuguacagggcuau...........    2          0          seq
    ...................................................cagcauuguacagggcuauGaaa.......    3          1          seq
```
